# Effects of Long-Term Space Flight on Erythrocytes and Oxidative Stress of Rodents

**DOI:** 10.1371/journal.pone.0032361

**Published:** 2012-03-07

**Authors:** Angela Maria Rizzo, Paola Antonia Corsetto, Gigliola Montorfano, Simona Milani, Stefania Zava, Sara Tavella, Ranieri Cancedda, Bruno Berra

**Affiliations:** 1 Dipartimento di Scienze Molecolari Applicate ai Biosistemi, DiSMAB, Università degli studi di Milano, Milan, Italy; 2 Università degli Studi di Genova & Istituto Nazionale per la Ricerca sul Cancro, Genova, Italy; Ohio State University, United States of America

## Abstract

Erythrocyte and hemoglobin losses have been frequently observed in humans during space missions; these observations have been designated as “space anemia”. Erythrocytes exposed to microgravity have a modified rheology and undergo hemolysis to a greater extent. Cell membrane composition plays an important role in determining erythrocyte resistance to mechanical stress and it is well known that membrane composition might be influenced by external events, such as hypothermia, hypoxia or gravitational strength variations. Moreover, an altered cell membrane composition, in particular in fatty acids, can cause a greater sensitivity to peroxidative stress, with increase in membrane fragility. Solar radiation or low wavelength electromagnetic radiations (such as gamma rays) from the Earth or the space environment can split water to generate the hydroxyl radical, very reactive at the site of its formation, which can initiate chain reactions leading to lipid peroxidation. These reactive free radicals can react with the non-radical molecules, leading to oxidative damage of lipids, proteins and DNA, etiologically associated with various diseases and morbidities such as cancer, cell degeneration, and inflammation. Indeed, radiation constitutes on of the most important hazard for humans during long-term space flights. With this background, we participated to the MDS tissue-sharing program performing analyses on mice erythrocytes flown on the ISS from August to November 2009. Our results indicate that space flight induced modifications in cell membrane composition and increase of lipid peroxidation products, in mouse erythrocytes. Moreover, antioxidant defenses in the flight erythrocytes were induced, with a significant increase of glutathione content as compared to both vivarium and ground control erythrocytes. Nonetheless, this induction was not sufficient to prevent damages caused by oxidative stress. Future experiments should provide information helpful to reduce the effects of oxidative stress exposure and space anemia, possibly by integrating appropriate dietary elements and natural compounds that could act as antioxidants.

## Introduction

Over the past 15 years space medicine has become increasingly concerned with the effects of spaceflight on hematological processes; astronauts have consistently returned from space-flight with a decreased red blood cell mass (RBC-M) “spaceflight anemia” and plasma volume (PV) [1], [2]. Although PV is known to be labile, current theories for the control of erythropoiesis cannot account for a decrease in RBC-M of 10% in less than 10 days. Erythrocytes exposed to microgravity have a modified rheology and undergo greater hemolysis [3]. We speculate that microgravity together with space radiation causes variations of cellular shape, plasma membrane composition, and peroxidative stress, which can be responsible of space anemia.

Hemorheologic variability, such as plasma viscosity, red cell aggregation and red cell deformability are of great importance for the passage of blood cells through the microcirculation. Cell membrane composition plays an important role in determining erythrocyte resistance to mechanical stress and it is well known that cell membrane composition is influenced by external events, such as hypothermia, hypoxia or gravitational strength variations.

The cell membrane is a lipid bilayer essentially formed by phospholipids, cholesterol and glycolipids. Small variations in percentage composition and molar ratio of the different classes of phospholipids and glycolipids, might induce changes in cell membrane's fluidity and permeability. Moreover this may also influence the activity of intrinsic membrane proteins, such as enzyme's and channels or ionic pumps. Finally, a different fatty acid composition of membrane components can result in a greater sensitivity to peroxidative stress, with a consequent increase in membrane fragility.

In the human organism, solar radiation or low wavelength electromagnetic radiations (such as gamma rays) from the Earth or space environment can split water to generate the hydroxyl radical, very reactive at the site of its formation, which can initiate chain reactions leading to lipid peroxidation. These reactive oxygen species (ROS) are shown to react with the non-radical molecules, leading to oxidative damage of lipids, proteins and DNA, causing various diseases and morbidities, including cancer, cell degeneration, and inflammation [4], [5].

In this view, radiation constitutes the most important hazard for humans during long-term space flights. Radiation protection is therefore mandatory to safeguard the well-being of future astronauts or crew members and to prevent the occurrence of future damages [6].

Antioxidant status reflects the dynamic balance between the antioxidant system of enzymes and molecules and the prooxidants that are constantly being generated. “Oxidative stress”, a more pronounced pro-oxidant state, resulting from a serious imbalance favoring oxidation, might be due to an excessive production of ROS, caused by exposure to toxics, radiations or pathological conditions, or from weakening of the antioxidant defense system.

The damage caused by ROS also includes DNA base alteration, which might cause permanent mutations, carbonyl modification of proteins, loss of sulfhydryl groups leading to inactivation of enzymes and increased proteolysis.

A number of defense mechanisms have been developed to protect the non-radical molecules from radical attack, thus limiting the damages. Several antioxidant enzymes can counteract the availability of ROS: superoxide dismutases (SOD), which transforms superoxide anion to hydrogen peroxide; glutathione transferases (GST) and glutathione peroxidases (GPx) [7]. These latter enzymes are associated with the detoxification reactions of xenobiotic compounds that have been activated to electrophilic molecules. The glutathione peroxidase removes hydrogen peroxide generated by the superoxide dismutase. There are also a number of repair enzymes that destroy free-radical-damaged proteins, DNA and oxidized fatty acids from peroxidized molecules. Other molecules contribute to the overall antioxidant defenses of the body against radical damage. They can be found both intra and extracellularly, including tocopherol, reduced glutathione, vitamin C, carotenoids, and urate. Alpha-tocopherol (vitamin E) occurs in membranes and lipoproteins and prevents the chain reaction of lipid peroxidation by scavenging intermediate peroxyl radicals. Vitamin C converts the vitamin E radical back to alpha-tocopherol. Reduced glutathione participates in a number of radical-scavenging reactions, mainly in the respiratory tract and red blood cells. Urate also scavenges free radicals, and beta-carotene functions as a general lipophilic antioxidant [8].

The Mice Drawer System (MDS) is an Italian Space Agency (ASI) facility developed by Thales-Alenia Space to support mice onboard the International Space Station during long-duration missions (from 100 to 150-days) [9]. It was launched with STS-128 on August 28^th^, 2009 and installed on the ISS. The leading experiment investigated the genetic mechanisms underlying bone mass loss in microgravity in two strains of mice: Wild type mice and pleiotrophin (PTN) transgenic mice. Additional information on the MDS experiment, the payload adopted and the mice strains utilized can be obtained from the companion article by (Cancedda et al). The present study is contributing to the investigation of the microgravity effects on the whole body through a tissue-sharing program (TSP) [10]. In particular, we performed the analysis of erythrocytes from mice housed in MDS for about 100 days on the ISS (MDS-ISS) and compared to mice housed in MDS payload replica on ground (MDS-ground) and mice housed under normal conditions (Vivarium). The aim of our research was to define the red blood cell membrane composition of the erythrocytes, to determine the oxidative stress that erythrocytes underwent. These data may be useful to design and start an applied research aimed at the identification of pharmacological, phytochemical or nutritional supplements against oxidative stress induced by space flight in astronauts.

## Results and Discussion

Table I reports the results of the analysis performed on the blood of mice that returned alive from space (MDS-ISS) and of the MDS-ground and vivarium controls. All 3 MDS-ISS mice have a higher erythrocyte concentration (RBC) and RDW% with a hematocrit near or above 50% compared to reference values and controls. Moreover PTN-2 and WT-2 MDS-ISS mice showed also a very high platelet concentration; on the contrary the Hb content did not show any alteration related to space flight.

**Table 1:** Hematological parameters of flown and control mice.

	REF.	PTN-1	PTN-2	WT-2	PTN-1	PTN-2	WT-2	PTN-1	PTN-2	PTN-3	WT-1	WT-2	WT-3
		MDS-ISS	MDS-ISS	MDS-ISS	MDS ground	MDS ground	MDS ground	Vivarium	Vivarium	Vivarium	Vivarium	Vivarium	Vivarium
**RBC** 106/ul	7.51–9.66	11.03	11.72	11.06	8.20	11.50	10.36	10.30	9.93	9.98	10.21	10.38	10.67
**HB** g/dl	12.8–16.1	15.8	16.5	16.0	11.8	16.8	16.0	16.3	16.1	17.0	16.2	16.4	17.3
**HCT** %	34–50	49.0	52.0	49.0	31.8	46.1	42.6	43.3	41.2	41.8	41.2	41.6	44.0
**MCV** fl	41–60	44	44	44	39	40	41	42	41	42	40	40	41
**MCH** pg	13–19	14.0	14.0	14.0	14.5	14.6	15.5	15.8	16.3	17.0	15.8	15.8	16.2
**MCHC**%	30–39	32.0	32.0	33.0	37.3	36.5	37.7	37.7	39.2	40.7	39.2	39.3	39.2
**RDW** %		18.0	18.7	17.5	11.6	13.0	12.6	14.2	14.8	14.0	13.4	12.3	13.3
**WBC** 10^3^/ul	4.5–9.1	5.7	4.1	3.4	4.9	1.4	3.1	5.2	4.2	5.7	4.7	6.2	5.3
**Neutrophils** %	21–57	26	23	18	18	30	24	22	24	38	18	10	8
**Eosinophils**%	0–3	8.0	4.0	4.0	1.0	0.0	0.0	0.0	0.0	0.0	1.0	0.0	0.5
**Basophils** %	0–3	3	2	1	0	0	0	0	0	0	0	0	0
**Linphocytes** %	49–82	59.0	66.0	73.0	80.0	70.0	75.0	77.5	75.0	61.5	80.0	89.5	91.0
**Monocytes** %	2–8	4.0	5.0	4.0	1.0	0.0	1.0	0.5	1.0	0.5	1.0	0.5	0.5
**Platelets** 10^3^/ul	421–773	694	1266	1475	289	705	990	634	717	1307	449	921	774
**MPV** fl		5.6	5.3	5.2	9.8	16.8	12.0	14.0	11.5	10.2	13.8	13.5	14.5
**PCT** %					0.285			0.889	0.822		0.618		
**PDW**%		26.2	24.2	22.1	11.9		22.2	19.5	20.2	13.5		20.7	0.0

REF. reference intervals are obtained from common normal inbred strains of mice.

These data are in agreement with observations made in humans where increased blood concentration are probably due to body fluid shift,renal function, and to a dysfunction of erythropoiesis. A highly random distribution of red cell width is also observed, probably due to a higher hemolysis [11].

Previous experiments, conducted during a 12 days space flight, showed an increase of RBC concentration and a parallel decrease of red blood cell distribution width (RDW) and mean corpuscle value (MCV) as result of water loss during space flight [12]. Our data from blood of animals, that remained in space for 3 months (MDS-ISS, PTN and WT), are different for what RDW is concerned indicating that the long duration microgravity exposure influenced the erythropoiesis and/or increased cell aging.

Oxidative stress has been correlated with several diseases and morbidities. Space radiation, microgravity and their effects on living organisms might generate oxidative stress responsible of space anemia [13]. The MDS mission offered to us the possibility, for the first time to measure in vivo oxidative stress after a long permanence of the animals on board the ISS.

For these reasons, we determined the Thiobarbituric Acid Reactive Substances (substances formed as byproducts of lipid peroxidation – TBARS) content as an indicator of oxidative stress and the activities of antioxidant enzymes in the mice erythrocytes.

**Figure 1:**
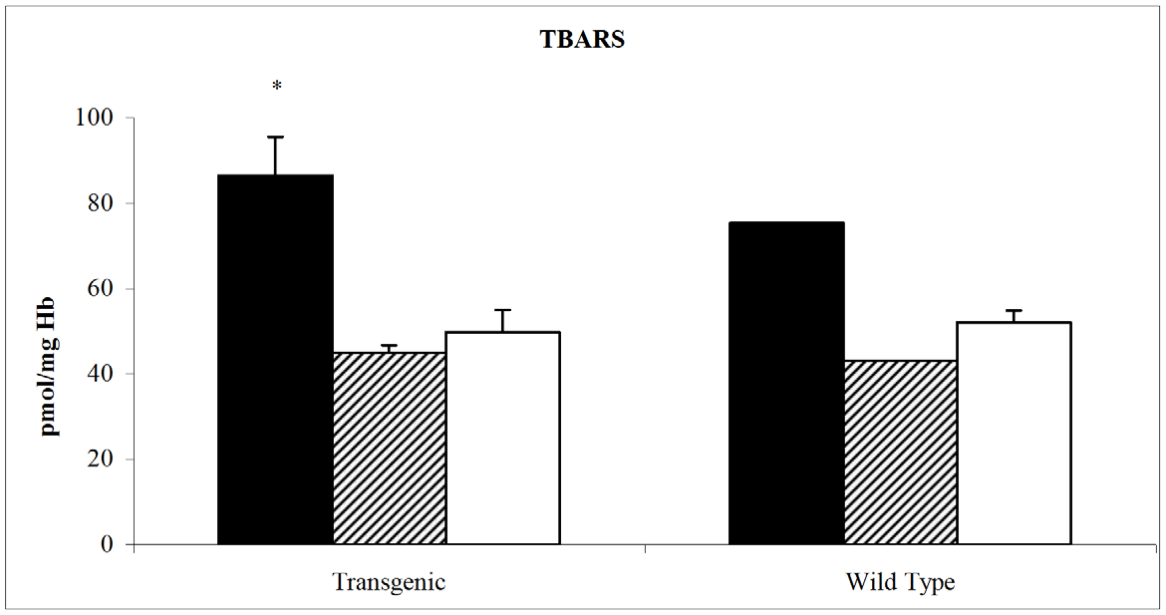
Thiobarbituric acid reactive substances (TBARS) content (pmol/mg Hb, mean± SD) in PTN and wild type mice erythrocytes after space flight. MDS-ISS (black bars), Ground MDS controls (line bars) and vivarium (white bars). * p<0.05 MDS-ISS vs Ground MDS control. Statistical analysis performed only on PTN mice.

We observed in the PTN MDS-ISS mice a statistically significant increase of TBARS in erythrocytes mice compared to both Earth controls (PTN MDS-ground and vivarium), figure 1. The same trend was observed for the wild type mice. These data indicated that during space flight mice underwent oxidative stress, which generated lipid peroxidation products such as malonildialdehyde.

**Figure 2:**
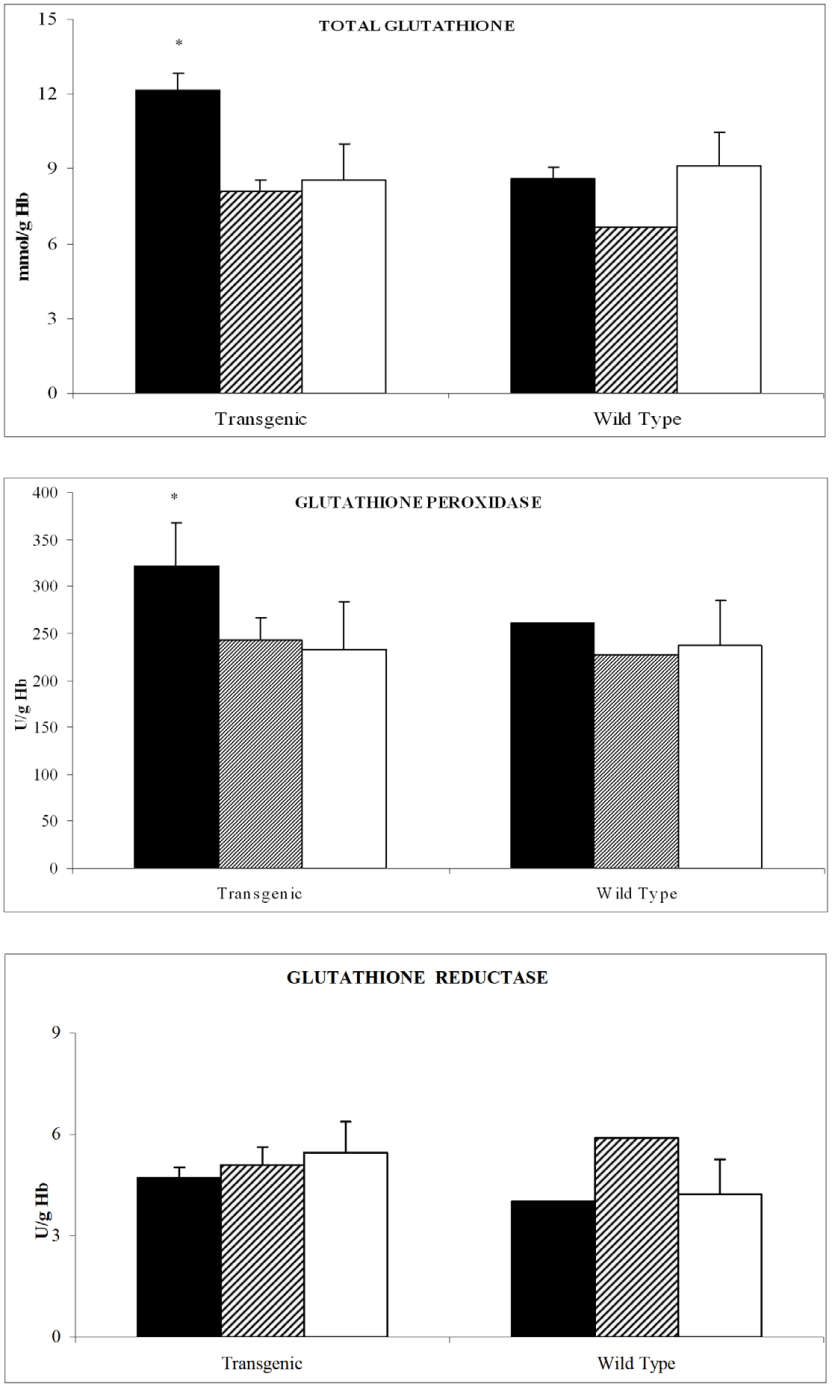
Antioxidant enzymes and glutathione content in PTN and wild type mice erythrocytes after space flight. **A:** Total glutathione (GSH) content (nmol/g Hb, mean+sd) **B:** glutathione peroxidase (GPx) (U/g Hb, mean+ SD) **C:** Glutathione reductase (GR) (U/g Hb, mean+ SD). MDS-ISS, (black bars), Ground MDS controls (line bars) and vivarium (white bars). * p<0.05 MDS-ISS vs Ground MDS control. Statistical analysis performed only on PTN mice.

By analyzing the content of erythrocyte glutathione, the major antioxidant present in these cells, we observed that the total content of this thiol was significantly increased after space flight compared to control in PTN MDS-ISS mice (Fig. 2). In addition, the enzyme involved in glutathione utilization, the glutathione peroxidase, which eliminates hydroperoxides from lipids, was significantly more active in PTN MDS-ISS mice. On the contrary, the activity of glutathione reductase, a very important enzyme that regenerates glutathione after its oxidation was not modified during the space flight.

Several antioxidant enzymes can limit the availability of ROS, including superoxide dismutases (SOD), which transforms superoxide anion to hydrogen peroxide, and Catalase, that removes hydrogen peroxide.

**Figure 3:**
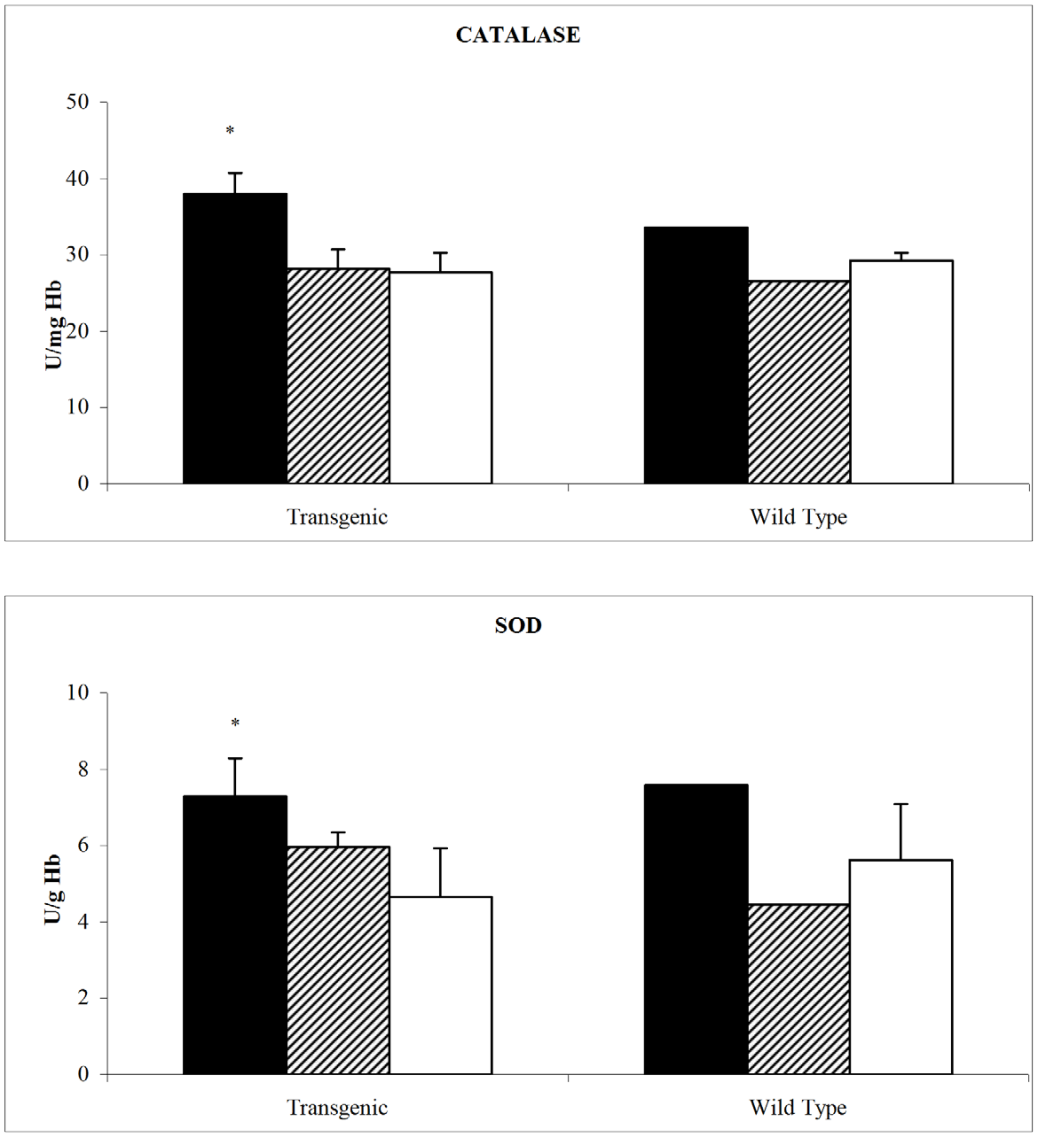
Catalase (Panel A, U/mg Hb+SD) and Superoxide dismutase (Panel B U/g HB+SD) activities in PTN and wild type mice erythrocytes after space flight. MDS-ISS (black bars), Ground MDS controls (line bars) and vivarium (white bars). * p<0.05 MDS-ISS vs Ground MDS control. Statistical analysis performed only on PTN mice.

Both enzymes had a significantly higher activity in flown PTN mice erythrocytes (Fig. 3). The same trend was observed for WT mice, even if no statistics may be performed on these data.

Moreover it seems that flown PTN mice reached an higher level of TBARS, GSH and GSH peroxidase after space flight; these may be related to an higher sensitivity of PTN mice to oxidative stress. In fact in primary culture of human osteoblasts from osteoporotic tissue it was recently demonstrated that a down regulation of PTN is paralleled by an up regulation of genes involved in response to reactive oxygen species probably due to increased exposure of osteoblasts to oxidative stress in osteoporotic tissue [14].

The increase of these enzymatic activities and of glutathione intracellular content may indicate an induction of these proteins and may be also correlated to the age of newly synthesized erythrocytes; in fact it is demonstrated that during RBC aging the level and the activity of these enzymes significantly decrease [15].

An induction of antioxidant enzymes has been reported in the liver of mice flown for 13 days in previous space missions [16]. Moreover, simulated microgravity and chronic stresses induced antioxidant responses in lymphocytes, brain and muscles [17]–[20].

Finally, we investigated the lipid composition of RBC membrane phospholipids. The membrane phospholipid content and composition of mice erythrocytes is reported in table II.

**Table 2:** Content of phospholipids and percentage distribution of each phopholipid class in cell membrane of mice erythrocytes.

	WILD TYPE			PTN		
	MDS-ISS	MDS-GROUND	VIVARIUM	MDS-ISS	MDS-GROUND	VIVARIUM
PL µg/mg HB	27.65	23.45	36.02±9.40	36.09±0.21*	28.33±4.57	44.97±16.43
% PE	30.83	30.71	36.65±0.76	38.14±4.19	36.16±2.21	31.92±6.77
% PI	1.93	6.48	4.750±0.61	3.41±1.61	6.82±4.35	4.74±1.56
% PS	27.72	9.70	12.01±1.85	19.74±2.71*	12.54±1.39	9.63±0.82
% PC	13.95	27.41	26.94±2.54	22.15±1.50*	28.16±1.83	27.42±6.08
% SM	25.57	25.71	19.64±0.88	16.56±1.37	16.32±2.57	26.28±10.68

(PE, phopshatidylethanolamine; PI, phopshatidylinositol; PS, phosphatidylserine; PC, Phosphatidylcholine; SM, sphingomyelin).

* = p<0.05 MDS-ISS vs MDS-Ground control.

As the lipid membrane composition may be greatly influenced by food quality and consumption [21] we first compared the fat composition of food bars used in the MDS facility during flight and ground control (produced by Mucedola, Italy) with the one of the standard food utilized for vivarium mice (Global Diet 2018, Harlan, Italy). As the fat content was different between the two diets, we considered for statistical comparison only the membrane lipid composition of MDS-ISS mice and MDS-ground control mice that had the same diet.

**Figure 4:**
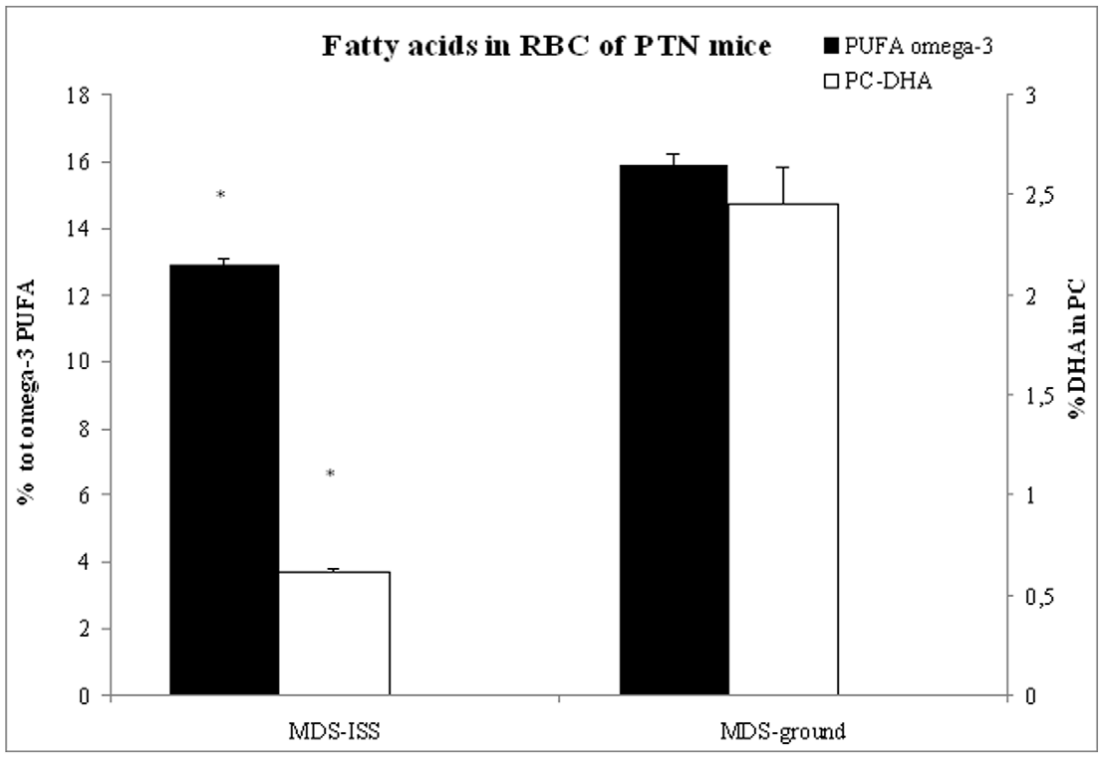
Total omega-3 fatty acid content (black bars) and phosphatidylcholine DHA content (white bars) in PTN mice erythrocytes after space flight (MDS-ISS, Ground MDS controls). * p<0.05 MDS-ISS vs Ground MDS control.

In the RBC membrane of MDS-ISS mice there was an increase of phospholipids, statistically significant for the PTN mice, with changes in their relative percentage composition. In particular, the content of phosphatidylcholine (PC) was significantly reduced, whereas the phosphatidyl serine content was increased in MDS-ISS PTN mice. Moreover, we purified the single phospholipid classes and analyzed the fatty acid composition of each phospholipid. We noticed a significant decrease of the total polyunsaturated fatty acids of the omega-3 series in PTN mice (from 15.39% in MDS-ground mice to 12.89%in MDS-ISS mice). In particular, in the purified PC the amount of docosaexahenoic acid (DHA) was significantly reduced from 2.45 to 0.62% (Fig. 4).

These data are in agreement with the increase of TBARS, which could have been generated from oxidative damage of membrane polyunsaturated lipids and may be related to changes in membrane fluidity of erythrocyte or may be the results of microgravity adaptation [3].

It is noteworthy that, in addition to structural roles, omega 3 fatty acids, especially DHA, play major roles in signal transduction and are increasingly being recognized as reservoirs of lipid messengers. Specific precursors are cleaved from membrane phospholipids, in particular from PC, upon stimulation by neurotransmitters, neurotrophic factors, cytokines, membrane depolarization, ion channel activation, etc.

Impaired polyunsaturated fatty acid (PUFA) status is observed, and measured in erythrocyte, in numerous non physiological states and chronic diseases, like heart disease, metabolic syndrome, rheumatoid arthritis, and other inflammatory conditions, pulmonary disorders, and some psychiatric disorders [22], [23].

There are several potential mechanisms that mediate the effects of PUFA on human health including antithrombotic and anti-arrhythmic effects, decreased heart rate variability and resting blood pressure, decreased serum low density lipoproteins (LDL) cholesterol and triglyceride content and increased insulin sensitivity. Other possible mechanisms of omega-3 PUFA are favorable effects on endothelial function, anti-inflammatory effects as well as neuroendocrine influences including modulation of the hypothalamic-pituitary-adrenocortical axis activity [24].

The operation of the space station and the human exploration will require long duration missions. However, mission duration will be limited by the degree of knowledge on the level of the physiological adaptation as well as on the acceptable limits of exposure to specific conditions during the permanence in space environment; our observations may be of particular importance for future manned long term space flight to prepare and implement a nutritional and nutraceutical program to maintain crew health.

## Materials and Methods

The Mice Drawer System (MDS) is an Italian Space Agency (ASI) facility, which is able to support mice onboard the International Space Station during long-duration exploration missions (from 100 to 150-days) by providing living space, food, water, ventilation and lighting.

Mice can be accommodated either individually (maximum 6) or in groups (4 pairs). MDS is integrated in the Space Shuttle middeck during transportation (ascent and descent) to the ISS and in an EXPRESS Rack in Destiny, US Laboratory during experiment execution.

MDS was launched on board of ISS with 3 wild type mice (C57BL/J10) and 3 transgenic mice with the Pleiotrophin (PTN), a growth and differentiation factor. The major aim was to study the genetic mechanisms underlying the bone mass pathophysiology and test the hypothesis that mice with an increased bone density are likely to be more protected from osteoporosis, when the increased bone mass is a direct effect of a gene involved in skeletogenesis (skeleton formation).).

For flight details refer to the paper of Cancedda et al. After flight, only 3 mice survived: 2 PTN transgenic mice and one wild type mouse.

Controls were run asynchronously in Genoa laboratories. In particular, 2 types of control were utilized. MDS ground control consisted in mice housed in a MDS payload replica on ground with the same profile of food, temperature, humidity and oxygen level. Vivarium mice, housed in normal conditions, constituted the second set of control mice. The three groups of mice will be hereafter referred as MDS-ISS, MDS-Ground and Vivarium. For additional information refer to the paper of Cancedda et al. (published in the same collection).

In all phases of the experiment (pre-flight, during the flight and post-flight) handling of animals was in accordance with the principles expressed in the “Guide for the care and the use of laboratory animals” (Office of Science and Health Reports of the USA National Institute of Health, Bethesda, USA). The approval of the MDS experiment was requested and obtained by the American Institutional Animal Care and Use Committee (IACUC protocol n° FLT-09-070 –Kennedy Space Centre) as well as by the Ethics Committee of the Animal Facility of the National Institute for Cancer Research (Genoa, Italy) and by the Public Veterinary Health Department of the Italian Ministry of Health (Ministero del Lavoro, della Salute e delle Politiche Sociali prot n° 4347-09/03/2009 DGSA.P.).

The main authors of this article were not directly involved in/responsible for designing and/or executing the animal maintenance part of the experiment. Instead they were allowed access to the mice at the end of the flight mission and of the ground control experiments and participated in the specific tissue collection. Additional information about the MDS hardware adopted for housing the animal in space, the animal behavior during the flight and the efforts made to reduce mice pain and suffering during the whole experiment are reported in the companion article by Cancedda et al.

### Blood Collection

Collection of blood of flight and ground mice was performed by retroorbital sinus puncture. Blood from each animal was divided into 2 microtubes containing EDTA, for hematocrit determination, and Li-heparin for erythrocyte separation from plasma respectively.

The heparin tubes after gentle agitation were centrifuged at 850 g at 4°C for 5 min. After centrifugation, plasma was immediately separated and frozen in dry ice.

The erythrocyte pellet was washed two times with 10 vol of cold PBS, subdivided into Eppendorf microcentrifuge tubes, and immediately frozen on dry ice. An aliquot of 15 µl of erythrocytes was freshly used to prepare glutathione extract. For this purpose, cells were homogenized on ice in 10% metaphosphoric acid, centrifuged at 13,000 rpm for 10 min and the supernatant was frozen on dry ice. Samples were transported from Kennedy Space Center (flight samples) and Genoa laboratories (ground control samples) to our laboratory in Milan as frozen samples in dry ice using a specialized courier.

### Laboratory of analysis for hematochemistry

Blood collected into the EDTA tubes was utilized for hematocrit count and standard laboratory analysis. Flight samples were analyzed by USA Miller laboratory, School of Medicine University of Miami, whereas ground control samples were analyzed in Genoa by a private veterinarian laboratory.

### RBC separation and analysis

Erythrocyte unsealed ghosts were prepared by hypotonic lysis (30 min, 4°C) in 40 vol of 5 mM NaHPO4 buffer pH 8.0. Membranes were washed in 20 vol of the same buffer and this process was continued until the ghosts were free of residual hemoglobin.

Cell hemolysates were obtained from washed cells by two cycles of freezing, unfreezing and centrifuging. Ghosts were used to assess cell membrane lipid composition (fatty acids, cholesterol and phospholipids) and lipid peroxidation as thiobarbituric acid-reactive substances (TBARS). The hemolysed fraction was tested for catalase, peroxidase, reductase and SOD enzyme activities.

### Lipid extraction and analysis

Ghosts were extracted with three different chloroform/methanol mixtures (1∶2, 2∶1, 1∶1 v/v), dried and partitioned with 1 vol of water and 2 vol of chloroform/methanol/water 3/48/47 (v/v/v) to obtain an organic and an aqueous phase which were both dried and resuspended in chloroform/methanol 2/1. Lipids were quantified as previously described [25].

### Enzyme assay

Hemoglobin was determined on cell lysate as previously described [26] and used to normalize enzyme determinations.

Superoxide Dismutase (SOD) activity assay: the enzyme activity was assayed on aliquots of the hemolysate fraction using the method based on inhibition of NAD(P)H oxidation, according to Paoletti and Mocali [27]. One unit of SOD activity is defined as the amount of enzyme required to inhibit the rate of NADPH oxidation by 50%.

Catalase (CAT) activity assay: the enzyme activity was assayed on aliquots of the hemolysate fraction measuring the consumption of H_2_O_2_ according to Aebi [28]. One unit of CAT activity is defined as the amount of enzyme required to catalyze the decomposition of 1 µmole H_2_O_2_ min-1.

Glutathione Reductase (GR) activity assay: the enzyme activity was assayed on aliquots of the haemolysate fraction following the oxidation of NADPH according to Pinto et al. [29].

Glutathione Peroxidase (GPx) activity assay: the enzyme activity of the selenium-dependent GPx was assayed on aliquots of the hemolysate fraction according to Prohaska and Ganther [30].

One unit of GR or GPx is defined as the amount of enzyme required to catalyze the oxidation of 1 µmole NADPH min-1.

Total Glutathione was assayed with an enzymatic method according to Griffith [31].

### TBARS (fluorimetric assay)

Samples of ghost extracts, standards (from 2.5 to 100 pmoli TEP, 1,1-3,3 tethraethoxypropane) and blank were assayed according to TBARS method [32] with and without stimulation with FeSO_4_ and ascorbic acid.

TBARS Fluorescence was quantified comparing samples with standard curve (Varian Carly Eclipse; excitation wavelength: 517 nm, emission wavelength: 550 nm).

### Statistics

Enzymatic assays were repeated at least 4 times. Data report mean ± S.D. values, t test was performed to compare flight (MDS-ISS) with ground controls (MDS-ground) and vivarium, only for PTN mice.
